# Perceptions of mental toughness in elite swimmers: A Q methodology study

**DOI:** 10.3389/fpsyg.2026.1756286

**Published:** 2026-01-21

**Authors:** Alay Kesler, Yasin Yıldız, Umut Sevilmiş, Filiz Küçükalpelli, Doğukan Batur Alp Gülşen

**Affiliations:** 1 Department of Coaching Training, Faculty of Sports Sciences, Istanbul University-Cerrahpasa, Istanbul, Türkiye; 2 Department of Recreation, Faculty of Sports Sciences, Aydın Adnan Menderes University, Aydın, Türkiye; 3 Department of Coaching Education, Faculty of Sports Sciences, Kilis 7 Aralık University, Aydın, Türkiye; 4 Department of Coaching Education, Faculty of Sports Sciences, Mehmet Akif Ersoy University, Aydın, Türkiye

**Keywords:** athlete, elite swimmers, mental toughness, Q method, sports psychology

## Abstract

This study aimed to understand the perceptions of mental toughness among elite swimmers. A qualitative research method, the Q methodology, was employed to delve into the individual perspectives of participants. Data were analyzed using the PQ Method software to identify participants’ perceptions of mental toughness. The study involved 23 elite swimmers (16 males and 7 females), all of whom had qualified for the Turkish National Swimming Championships. Participants represented a range of swimming disciplines, including freestyle, breaststroke, butterfly, and backstroke. The findings indicate that participants grouped their views on mental toughness into three main factors. The first factor reflected a determined attitude towards overcoming challenges and achieving goals. The second emphasized the importance of learning from mistakes and valuing feedback from coaches. The third revealed athletes’ ability to stay calm under pressure, alongside their sensitivity to coaches’ evaluations. These three distinct perspectives demonstrate that mental toughness is not a singular concept but rather a multidimensional construct influenced by personal resilience, interpersonal dynamics, and emotional regulation. Together, these viewpoints indicate that mental toughness is represented through multiple psychological and relational dimensions within this sample.

## 1 Introduction

In recent years, the mental health and psychological well-being of elite athletes have been increasingly recognized as critical components of overall athletic success (Reardon et al., 2019). Elite-level competitive athletes are continuously exposed to high performance pressure, ongoing evaluation, and demanding training environments. All of these factors require effective psychological regulation. When athletes perceive competitive demands as overwhelming or uncontrollable, they may experience increased stress, impaired concentration, and maladaptive coping responses (Nicholls et al., 2012; Yildiz et al., 2025). Within this context, mental toughness has attracted considerable attention as a psychological resource that enables athletes to maintain high-level performance in the face of adversity.

Mental toughness is commonly defined as an athlete’s capacity to remain confident, focused, and determined while effectively regulating emotions and behavior in challenging situations (Gucciardi et al., 2015; Jones et al., 2007). Despite broad consensus regarding its importance, mental toughness remains a conceptually contested construct. Early work in the field conceptualized mental toughness primarily as a trait-like disposition, emphasizing relatively stable characteristics such as confidence, persistence, and control (Clough et al., 2002; Jones et al., 2007). From this perspective, mentally tough athletes are viewed as consistently resilient individuals who demonstrate superior coping and performance stability across contexts.

Within this context, mental toughness (MT) has emerged as a key construct in sport psychology. Mental toughness refers to an athlete’s ability to remain determined, focused, confident, and in control under pressure, providing a psychological edge over opponents (Gucciardi et al., 2015; Guillén and Laborde, 2014). Numerous studies have provided empirical evidence demonstrating that mental toughness is one of the most critical psychological attributes linked to athletic success (Connaughton et al., 2010; Gucciardi, 2017; Jones et al., 2010).

The coexistence of differing perspectives highlights a significant issue within the mental toughness (MT) literature. Although the concept is widely used and its importance is frequently emphasized, there is limited consensus regarding how athletes themselves understand and prioritize mental toughness. Existing research has predominantly relied on variable centered methodologies, such as psychometric scales and regression based models, which assume a common underlying structure of MT across individuals (Sheard et al., 2009; Gucciardi et al., 2015). While these approaches have contributed valuable insights, they may obscure individual differences in meaning making and overlook how athletes subjectively organize psychological attributes into coherent personal frameworks.

This characteristic becomes even more essential in individual sports, where athletes compete and cope largely on their own. Swimming, by its very nature, is a predominantly individual sport, which may increase athletes’ vulnerability to mental health issues compared to those participating in team sports (Meggs and Chen, 2018; Pluhar et al., 2019). In such a setting, swimmers must rely not only on physical competence but also on a high degree of psychological resilience to manage the mental demands of training, competition, and self-evaluation. Previous research has shown that mental toughness plays a significant role in enhancing swimming performance (Beattie et al., 2019; Hirsch et al., 2024).

Most studies exploring mental toughness have employed either qualitative interviews or validated psychometric instruments, generating valuable insights into its structure and influence (Crust and Clough, 2011; Morrison et al., 2024; Yarayan et al., 2018). However, there is a noticeable lack of research integrating both qualitative and quantitative perspectives, particularly those that center on athletes’ own perceptions and lived experiences (Juan and Lopez, 2015; Nicholls et al., 2009). This gap limits our understanding of the contextual and subjective nature of mental toughness.

To address this gap, research designs that center athletes’ subjective perspectives are needed. Q methodology offers a systematic and theoretically appropriate approach for examining such perspectives. By integrating qualitative expression with quantitative factor analytic procedures, Q methodology enables the identification of shared viewpoints while preserving the subjective structure of individual meaning systems (Brown, 1980; Ramlo, 2016). Unlike traditional survey methods, Q methodology does not impose predefined dimensional structures but instead reveals how participants prioritize and relate concepts within a given domain.

Applying Q methodology to the study of mental toughness allows for a person centered exploration of the construct, addressing longstanding calls for methodological diversity and conceptual clarity in the field (Gucciardi et al., 2017). Rather than asking whether athletes possess more or less mental toughness, this approach examines what mental toughness means to them, how its elements are organized, and where viewpoints converge or diverge.

### 1.1 The present study

Given the absence of a single, universally accepted theoretical framework for mental toughness, the present study adopts a pluralistic and contextual perspective, drawing primarily on the performance based and ecological conceptualizations of mental toughness (Connaughton et al., 2010; Connaughton et al., 2008; Jones et al., 2002; Gucciardi et al., 2017). In this respect, mental toughness is conceptualized not as a fixed personality trait, but as a context sensitive psychological capacity manifested through distinct subjective profiles, which aligns well with the epistemological assumptions of Q methodology.

The present study employed Q methodology to explore how elite swimmers conceptualize mental toughness. By identifying shared viewpoints among athletes, the study aims to contribute to a more nuanced and context sensitive understanding of mental toughness in elite sport. Specifically, the study addresses the following research questions:

*RQ1*: What distinct viewpoints regarding mental toughness emerge among elite swimmers?

*RQ2*: Which statements differentiate these viewpoints, and which reflect shared consensus?

*RQ3*: How do these viewpoints relate to prominent theoretical components discussed in the mental toughness literature, including persistence, emotional regulation, and relational dynamics?

By adopting a person centered methodological framework, this study seeks to extend existing mental toughness research beyond variable-centered models and provide a theoretically grounded understanding of how elite swimmers perceive and interpret this construct. (Yarayan et al., 2018; Yarayan and İlhan, 2018).

## 2 Method

In this study, the Q methodology, which encompasses both quantitative and qualitative processes, was employed to elucidate elite swimmers’ perceptions of mental toughness. Q methodology is a research method that allows for the subjective measurement of an individual’s perspective, opinion, belief, behavior, and attitude (Brown, 1980). One of the aims of Q methodology is not to expect participants to directly confirm a predetermined assumption or prediction. Instead, participants are asked to express their own views by considering the given Q statements (Karasu and Peker, 2019). In this way, the Q methodology seeks to address how subjective the research conducted is (Ramlo, 2016). One of the most significant contributions of the Q methodology to research is its ability to determine whether participants converge on a common viewpoint, and if so, to establish the direction of that viewpoint and prioritize its importance (Yılmaz, 2021).

In the Q method, the qualitative part consists of the statements collected from participants, while the quantitative part constitutes the factor analysis conducted. This factor analysis is used to group the Q sorts that converge under common views, forming a fundamental aspect of the Q methodology (Ramlo, 2016).

### 2.1 Research model

In this study, Q methodology was utilized to elucidate elite swimmers’ perceptions of mental toughness. Q technique, commonly employed in psychology and social sciences, combines the strengths of both quantitative and qualitative methods and involves data analysis using the PQMethod - 2.35 software (Demir and Kul, 2011). This research was conducted in compliance with the principles outlined in the Helsinki Declaration.

### 2.2 Study group

Although Q methodology typically recommends a sample size between 40 and 60 participants, it is still possible to conduct robust research with a smaller number of participants from diverse backgrounds relevant to the subject matter (Shinebourne, 2009; Watts and Stenner, 2005). The inclusion criteria for the study were being 18 years of age or older, actively participating in swimming competitions, and competing at the national level. Athletes invited to the study were included after providing informed voluntary consent. Participants were informed that they could withdraw from the study voluntarily at any stage of the research process without any consequences.

### 2.3 Data collection instruments

In this study, a research form was developed to collect data regarding athletes’ perceptions of mental toughness. Initially, a comprehensive literature review was conducted to identify the core components of mental toughness and to guide the development of the Q statements. Following the literature review, the process of developing Q sort statements was initiated by the researchers. In generating the statements, established scales in the mental toughness (MT) literature and expressions derived from previous qualitative studies were taken into consideration. In addition, interviews were conducted with three researchers who have prior experience and publications in the field of MT, resulting in an initial pool of 45 statements. To ensure content validity, all statements were evaluated by two experienced coaches and three experts working in the field of sport psychology. Beyond subject matter experts, a language specialist reviewed the statements to assess their linguistic clarity and appropriateness. Based on the recommendations of all experts and the consensus of the researchers, 15 statements were removed due to their limited relevance to the research purpose, semantic overlap with other statements, or the potential for ambiguous interpretation. Following this process, 30 statements that best represented the construct of mental toughness were selected and finalized as the Q set. The final form also included two open-ended questions asking participants to explain their reasons for placing specific statements at the extreme ends of the Q sorting distribution (negative pole: −4; positive pole: +4).

### 2.4 Items included in the research form

See Table 1.

**Table 1:** Q method statements and numbers related to dimensions of mental toughness.

1.	Fear of making mistakes often worries me.
2.	Making mistakes frequently encourages me to be more careful.
3.	I feel bad when I make mistakes during a competition.
4.	When I make mistakes during a competition, it encourages me to focus more and learn from my mistakes.
5.	Instead of giving up when faced with difficulties, I make an effort to solve problems.
6.	I tend to give up quickly when faced with difficulties.
7.	It’s challenging for me to improve my performance when I start a competition poorly.
8.	I can improve my performance by increasing my concentration when I start a competition with difficulty.
9.	I get worried about my coach’s thoughts on my performance when I make mistakes.
10.	Feedback from my coach increases my focus and motivates me to improve my performance when I make mistakes.
11.	I perform at my best under pressure.
12.	Under pressure, my confidence in my abilities decreases, and the fear of failure affects me.
13.	I get too nervous to showcase my full potential.
14.	I turn potential threats into opportunities.
15.	I am a confident athlete with high self-esteem.
16.	I struggle to build confidence in myself in a sports environment.
17.	I can keep myself calm under pressure.
18.	I struggle to cope with stress under pressure.
19.	I am afraid to take responsibility during critical moments of a competition.
20.	I do my best during critical moments of a competition.
21.	I can easily keep up with a high-intensity training pace.
22.	It’s difficult for me to keep up with high-intensity training.
23.	I am determined to complete the tasks I need to do.
24.	I struggle to complete the tasks I need to do.
25.	I have clear goals that are important to me.
26.	I struggle to deal with uncertainties in life.
27.	I am a highly motivated athlete.
28.	I am a low-motivation athlete.
29.	When I experience failure or disappointment, I learn from those experiences and focus on my goals.
30.	When I experience failure or disappointment, I give up on my goals.

### 2.5 Data collection and analysis

Following the development of the form, the data collection process commenced. In this phase, each statement on the form was transcribed onto separate cards and distributed to the athletes. Participants were instructed to read the statements and arrange them on the Q sort grid presented by the researcher according to their level of agreement with each item. The placements and rankings made by the athletes were duly recorded.

Upon completion of data collection, the analysis phase was initiated. Initially, a correlation matrix of the participants’ Q sorts was computed to reflect the degree of similarity between their subjective rankings (Brown, 1980). Based on this correlation matrix, factor analysis was conducted. The Principal Component Analysis (PCA) method was employed for factor extraction, with the significance threshold value set at 0.47, calculated using the formula 2.58 × 1/√30.

To enhance the interpretability of the factor structure, Varimax rotation was applied. Factor rotation is a procedure used to examine clusters of similar Q sorts and determine which perspectives predominate among participants (McKeown and Thomas, 2013). For each factor, normalized weighted average scores of the Q statements, known as Z scores, were calculated based on the responses of participants who significantly loaded on that factor. These Z scores serve as the foundation for interpreting the distinctive characteristics of each factor (Van Exel and De Graaf, 2005).

As a result of the analyses, a three-factor solution was adopted to provide statistically and theoretically robust data interpretation.

## 3 Findings

See Table 2.

**Table 2:** Demographic information of participants.

Demographic variables		ƒ	%
Gender	Male	16	69,6
	Female	7	30,4
Swimming style	Freestyle	9	39,1
	Backstroke	2	8,7
	Breaststroke	10	43,5
	Butterfly	2	8,7
Receiving assistance from a sports psychologist	Yes	2	8,7
	No	21	91,3
Total		23	100

When Table 3 is examined, it is seen that the data obtained from the principal component analysis with varimax rotation shows that 23 participants are grouped into 3 factors. It was determined that 11 of the athletes participating in the study were in factor 1, 8 athletes were in factor 2, and 4 athletes were grouped in factor 3. To clearly indicate which groups the athletes belong to, they are marked in bold in the table. It can be stated that 11 of the 23 athletes participating in the study (26% of the group) being gathered under the same factor indicates a general characteristic of the group. From this, it can be inferred that the mental toughness views of the athletes show a significant degree of similarity.

**Table 3:** Factor loadings of participants on mental toughness.

Participants	Factor 1	Factor 2	Factor 3
1	0.3029	**0.7900X**	0.2092
2	**0.6672X**	0.3404	0.2397
3	0.5396	**0.6196X**	0.4025
4	0.1436	**0.5231X**	0.2757
5	0.4092	**0.5523X**	0.2148
6	**0.5012X**	0.3475	0.4675
7	**0.7128X**	0.4328	0.0485
8	**0.5339X**	0.3064	0.3252
9	−0.1891	**0.7850X**	0.1257
10	**0.7155X**	0.0487	0.4162
11	0.5539	**0.6960X**	0.0036
12	**0.5612X**	0.3713	0.4316
13	**0.7540X**	−0.0317	0.1872
14	0.4729	0.0017	**0.6591X**
15	0.4805	**0.6292X**	0.2769
16	**0.6208X**	0.4004	0.3525
17	0.1422	0.1896	**0.7997X**
18	0.1412	0.4366	**0.6761X**
19	0.4078	0.4415	**0.6252X**
20	**0.5072X**	0.4352	0.3542
21	0.5048	**0.5424X**	0.4645
22	**0.6089X**	0.1674	0.5172
23	**0.5791X**	0.5332	0.1806

In Table 4, the correlation coefficients between the factors are provided. A low correlation between the factors indicates that the factors are diverging from each other, while a high correlation indicates that the factors are close to each other. Upon examining the table, it was determined that there is a high positive correlation between factor 1, factor 2, and factor 3. Additionally, there is a moderate positive correlation between factor 2 and factor 3. The presence of moderate and high levels of correlation indicates that the factors are closely related to each other.

**Table 4:** Correlation between factors.

Factors	Factor 1	Factor 2	Factor 3
Factor 1	1	0.7332	0.7132
Factor 2	0.7332	1	0.6372
Factor 3	0.7132	0.6372	1

Table 5 contains the mental toughness views of elite swimmers grouped under 3 factors. Upon examining the table, it is observed that the statement most agreed upon by the 11 athletes grouped under Factor 1 is *“When faced with challenges, instead of giving up, I strive to solve the problems.”* Conversely, the statement they most disagreed with is *“I am an athlete with low motivation.”*

**Table 5:** Z scores of items for factors and Q sort rankings.

Distinguishing statements regarding mental toughness	Factor 1		Factor 2		Factor 3	
	Z score	Q Sort Rankings	Z score	Q Sort Rankings	Z score	Q Sort Rankings
5. Instead of giving up when faced with challenges, I make an effort to solve problems.	1.624	4	0.928	3	0.854	2
29. When experiencing failure or disappointment, I learn from these experiences and focus on my goals.	1.504	4	−0.190	0	1.968	4
25. I have clear goals that are important to achieve.”	1.491	3	0.307	0	0.210	0
27. I am a highly motivated athlete.	1.120	3	1.509	3	0.212	0
4. When I make a mistake during a competition, it encourages me to focus more and learn from my mistakes.	1.107	3	0.789	2	0.552	1
20. I do my best during critical moments of a competition.	1.074	2	1.606	4	0.790	2
23. I am determined to fulfill the tasks I need to do.	1.001	2	0.784	2	0.841	2
15. I am a confident athlete with high self-esteem.	0.949	2	1.444	3	1.621	4
2. Making mistakes frequently encourages me to be more careful.	0.771	1	0.294	0	1.175	3
14. I turn potential threats into opportunities.	0.694	1	0.638	1	0.098	0
11. I do my best under pressure.	0.677	1	0.330	1	0.122	0
8. When I have a tough start to a competition, I can improve my performance by increasing my concentration.	0.642	1	0.424	1	0.645	1
10. When I make a mistake, my coach’s feedback increases my focus and I make an effort to improve my performance.	0.530	0	1.865	4	0.074	0
21. Adapting to high-intensity training pace is easy for me.	0.162	0	0.662	2	0.209	0
17. I can keep myself calm and composed under pressure.	0.049	0	−0.212	−1	1.136	3
12. Under pressure, my confidence in my abilities decreases, and the fear of failure affects me	−0.274	0	−0.629	−1	0.319	1
16. I struggle to build confidence in the sports environment.	−0.311	0	−0.916	−2	−1.530	−4
3. I feel bad when I make a mistake during a competition.	−0.507	0	0.591	1	0.052	−1
26. I struggle to deal with uncertainties in life.	−0.511	−1	−1.423	−4	−1.372	−4
22. It’s difficult for me to keep up with high-intensity training.	−0.654	−1	−0.883	−1	−1.284	−3
9. When I make a mistake, my coach’s thoughts about my performance worry me.	−0.756	−1	−0.012	0	0.860	3
18. I struggle to cope with stress under pressure.	−0.813	−1	−0.995	−1	−1.305	−3
7. It’s challenging for me to improve my performance when I start a competition poorly.”	−0.889	−2	−0.281	−1	0.774	1
24. I struggle to fulfill the tasks I need to do.	−1.076	−2	−1.088	−3	−1.201	−1
30. When I experience failure or disappointment, I give up on my goals.	−1.151	−2	−1.342	−3	−1.260	−3
1. Making mistakes frequently worries me.	−1.157	−3	0.103	0	−0.399	−1
13. I get too nervous to unleash my full potential.	−1.190	−3	−0.036	0	−1.207	−2
19. I fear taking responsibility during critical moments of a competition.	−1.191	−3	−1.356	−3	−1.210	−2
6. I give up and quit immediately when faced with challenges.	−1.323	−4	−1.982	−4	−0.533	−1
28. I am a low-motivation athlete.	−1.593	−4	−0.929	−2	−1.212	−2

For the 8 athletes grouped under Factor 2, the statement most agreed upon is *“When I make a mistake, my coach’s feedback increases my focus, and I strive to improve my performance.”* Conversely, the statement they most disagreed with is *“When faced with challenges, I immediately give up and quit.”*

For the 4 athletes grouped under Factor 3, the statement most agreed upon is *“When I experience failure or disappointment, I learn from these experiences and focus on my goals.”* Conversely, the statement they most disagreed with is *“I struggle to build confidence in the sports environment.”*

Table 6 contains the Q sort values and Z scores for the items that distinguish the 3 factors from each other. Upon examining the table, it was found that 8 items showed significant differences at the *p* < 0.01 level. Athletes grouped under Factor 1 showed higher participation in items such as *“Instead of giving up when faced with challenges, I strive to solve the problems (5)”* and *“I have clear goals that are important to achieve (25)”* compared to athletes in Factor 2 and Factor 3. It was observed that athletes in Factor 1 remained neutral while athletes in Factors 2 and 3 did not agree with the statement *“I struggle to build confidence in the sports environment (16).” Additionally, athletes in Factor 1 showed less participation in items such as “I struggle to deal with uncertainties in life (26),” “Mistakes I make worry me about my coach’s thoughts on my performance (9),” “Starting a competition poorly makes it challenging for me to improve my performance (7),” “Making mistakes often worries me (1),”* and *“I immediately give up and quit when faced with challenges (6)”* compared to athletes in Factors 2 and 3.

**Table 6:** Discriminating items for factor 1.

Q statements regarding mental toughness	Factor 1		Factor 2		Factor 3	
	Z score	Q sort rankings	Z score	Q sort rankings	Z score	Q sort rankings
5. Instead of giving up when faced with challenges, I make an effort to solve problems.	1.62*	4	0.93	**3**	0.85	**2**
25. I have clear goals that are important to achieve.	1.49*	3	0.30	**0**	0.21	**0**
16. I struggle to build confidence in the sports environment.	−0.31*	0	−0.92	**−2**	−1.53	**−4**
26. I find it difficult to cope with uncertainties in life.	−0.51*	−1	−1.42	**−4**	−1.37	**−4**
9. When I make a mistake, my coach’s thoughts about my performance worry me.	−0.76*	−1	−0.01	**0**	0.86	**3**
7. When I start a competition poorly, it’s challenging for me to improve my performance.	−0.89*	−2	−0.28	**−1**	0.77	**1**
1. Making mistakes frequently worries me.	−1.16*	−3	0.10	**0**	−0.40	**−1**
6. When I encounter difficulties, I immediately give up and quit.	−1.32*	−4	−1.98	**−4**	−0.53	**−1**

*p < .01.

Table 7 contains the Q sort values and Z scores for the items that distinguish the 3 factors from each other. Upon examining the table, it was found that 6 items showed significant differences at the *p* < 0.01 level. Athletes grouped under Factor 2 showed a positive approach to the statement *“When I make a mistake, my coach’s feedback increases my focus, and I strive to improve my performance (10)”* while athletes in Factors 1 and 3 remained neutral towards this statement. Additionally, athletes in Factor 2 showed a more positive approach to the statement *“I do my best in the critical moments of the competition (20)”* compared to athletes in Factors 1 and 3. For the statement *“Mistakes I make worry me about my coach’s thoughts on my performance (9),”* athletes in Factor 1 had a negative approach, athletes in Factor 3 had a positive approach, and athletes in Factor 2 remained neutral. For the statement *“I get too nervous to really show my potential (13),”* athletes in Factors 1 and 3 showed a negative approach while athletes in Factor 2 showed a neutral participation. For the statement *“When I experience failure or disappointment, I learn from these experiences and focus on my goals (29),”* athletes in Factors 1 and 3 showed a positive approach while athletes in Factor 2 showed a neutral participation. Regarding the statement *“I immediately give up and quit when faced with challenges (6),”* athletes in Factor 2 showed less participation compared to athletes in Factors 1 and 3.

**Table 7:** Discriminating items for factor 2.

Q statements regarding mental toughness	Factor 1		Factor 2		Factor 3	
	Z score	Q sort rankings	Z score	Q sort rankings	Z score	Q sort rankings
10. When I make a mistake, my coach’s feedback increases my focus and I make an effort to improve my performance.	0.53	**0**	1.87*	**4**	0.07	**0**
20. I do my best during critical moments of the competition.	1.07	**2**	1.61*	**4**	0.79	**2**
9. When I make a mistake, my coach’s thoughts about my performance worry me.	−0.76	**−1**	−0.01*	**0**	0.86	**3**
13. I get so nervous that I cannot really show my potential.	−1.19	−3	−0.04*	**0**	−1.21	**−2**
29. When I experience failure or disappointment, I learn from those experiences and focus on my goals.	1.50	4	−0.19*	**0**	1.97	**4**
7. When I start a competition poorly, it’s challenging for me to improve my performance.	−0.89	**−2**	−0.28*	**−1**	0.77	**1**
6. When I encounter difficulties, I immediately give up and quit.	−1.32	−4	−1.98*	**−4**	−0.53	**−1**

*p < .01.

Table 8 contains the Q sort values and Z scores for the items that distinguish the 3 factors from each other. Upon examining the table, it was found that 6 items showed significant differences at the *p* < 0.01 level. Athletes grouped under Factor 2 showed a more positive approach to the statements *“I can keep myself calm and composed under pressure (17),” “Mistakes I make worry me about my coach’s thoughts on my performance (9),”* and *“Starting a competition poorly makes it challenging for me to improve my performance (7)”* compared to athletes in Factors 1 and 3. For the statement *“I am a highly motivated athlete (27),”* athletes in Factors 1 and 2 showed a positive approach while athletes in Factor 3 remained neutral in their participation.

**Table 8:** Discriminating items for factor 3.

Q statements regarding mental toughness	Factor 1		Factor 2		Factor 3	
	Z score	Q sort rankings	Z score	Q sort rankings	Z score	Q sort rankings
17. I can keep myself calm and composed under pressure.	0.05	**0**	−0.21	**−1**	1.14*	**3**
9. When I make a mistake, my coach’s thoughts about my performance worry me.	−0.76	**−1**	−0.01	**0**	0.86*	**3**
7. When I start a competition poorly, it’s challenging for me to improve my performance.	−0.89	**−2**	−0.28	**−1**	0.77*	**1**
27. I am a highly motivated athlete.	1.12	**3**	1.51	**3**	0.21*	**0**
6. When I encounter difficulties, I immediately give up and quit.	−1.32	−4	−1.98	**−4**	−0.53*	**−1**

*p < .01.

Table 9 shows the items where consensus was achieved among Factor 1, Factor 2, and Factor 3. Accordingly, it is understood that athletes had the same thoughts on two items and showed positive participation in these two items. Athletes with a common mindset expressed that they are conscientious (Karasu and Peker, 2019) and that they strive to focus their concentration on adverse situations to achieve better results (Cranmer and Myers, 2015).

**Table 9:** Consensus items.

Q statements regarding mental toughness	Factor 1		Factor 2		Factor 3	
	Z score	Q sort rankings	Z score	Q sort rankings	Z score	Q sort rankings
23. I am determined to fulfill the tasks I have to do.	1.001	2	0.784	2	0.841	2
8. When I start a competition with a difficult beginning, I can improve my performance by increasing my concentration.	0.642	1	0.424	1	0.645	1

### 3.1 The qualitative findings regarding the items included in the research instrument

Two open-ended questions were directed to the participants regarding their ranking on the Q sort grid. Participants were asked to explain why they placed sentences in the extreme ends of the grid (+4: agree and −4: disagree).

Participants’ Opinions Regarding the +4 Extreme Value.

*"I have set goals for myself and I am doing my best to achieve them. When faced with difficulties, I do not give up; instead, I exert intense effort to solve the problems and reach a solution." (P22)*

*"I consider my mistakes to be invaluable experiences. Therefore, when I make mistakes, I do not stress out; instead, I learn from them." (P10)*

*"During training or competition, if I make any mistakes, I strive to do my best to improve my performance when my coach advises me." (P8)*

*"I have been actively involved in swimming since a young age. I always strive to do my best. Even if my motivation wavers, I push my limits until I achieve the best possible outcome. For this reason, even in the critical moments of a competition, I strive to do my best." (P19)*

*"I gave my best both in training and during competitions. As a result, I achieved the Turkish championship in my age category. Therefore, my confidence in myself is complete." (P2)*

Participants’ Opinions Regarding the −4 Extreme Value.

*"The athlete never gives up; instead, they are always ready to fight under any circumstances." (P3)*

*"Mentally, I always focus on success. Therefore, I always make an effort to keep my motivation high." (P20)*

*"I do not struggle with uncertainty; instead, I persistently fight until I find a solution to uncertainty." (P11)*

*"My confidence in my athleticism is boundless. Therefore, I have no difficulty in building confidence." (P18)*

## 4 Discussion and conclusion

Beyond identifying distinct viewpoints, the present findings make a theoretical contribution by demonstrating that mental toughness cannot be adequately explained by a single dominant model. Instead, the results suggest that trait-oriented, process oriented, and relational conceptualizations coexist within the same performance context. This plurality refines existing mental toughness frameworks by indicating that no single model fully captures how athletes interpret and prioritize psychological resources. Consequently, mental toughness should be theorized as a context-sensitive construct that accommodates multiple, equally meaningful conceptual pathways.

This study sought to explore how elite swimmers conceptualize mental toughness using Q methodology. The findings demonstrate that mental toughness is not experienced as a singular or uniform construct, but rather as a multidimensional and context sensitive phenomenon shaped by individual priorities, relational dynamics, and emotional regulation processes. As a result of this study, it was found that elite swimmers’ perceptions of mental toughness were categorized under three distinct factors; however, they commonly converged on key strengths such as coping with challenges, goal orientation, and openness to coach feedback. Eleven participants were grouped under Factor 1, eight under Factor 2, and four under Factor 3. The fact that 11 out of 23 participants were grouped together indicates a general characteristic of the group.

### 4.1 Factor 1: mental toughness as persistence and goal commitment

First factor participants emphasized their determination to overcome challenges and their commitment to solving problems rather than giving up, aiming to achieve their set goals. This mindset among the participants indicates a resilient and determined attitude in overcoming obstacles. This perspective aligns closely with trait-oriented models of mental toughness, which emphasize commitment, confidence, and control as core components of resilient performance (Clough et al., 2002; Jones et al., 2007). Numerous researchers have highlighted the significance of mental toughness among athletes in achieving high-level performance (Gould et al., 1987; Jones et al., 2007; Bull et al., 2005). Individuals with high mental toughness are characterized as being in control, determined, competitive, self-motivated, and capable of maintaining focus under pressure. Moreover, they view unexpected changes as opportunities for growth and remain undeterred in the face of uncertainty, knowing how to cope with challenges. On the other hand, individuals with low mental toughness tend to give up in uncertain situations and struggle to resist unexpected events (Luthans, 2002; Crust and Clough, 2005; Crust, 2007; Güngörmüş et al., 2015).

### 4.2 Factor 2: mental toughness as a relational and developmental process

Participants in the second factor expressed that when they make mistakes, the feedback from their coaches increases their focus and helps improve their performance. They also mentioned putting in extra effort to perform at their highest level even during critical moments of a competition. This mindset among the participants highlights the importance they place on their coaches’ guidance and feedback in enhancing their performance. This viewpoint provides strong support for process oriented and developmental models of mental toughness (Connaughton et al., 2010; Gucciardi, 2017), which argue that mental toughness evolves through experience, feedback, and environmental interactions. Based on the existing literature, athletes have reported that coaches and sport psychologists play a significant role in enhancing mental toughness (Tunç et al., 2018; Yarayan et al., 2018). Coaches are crucial in ensuring athletes maintain an optimal and stable psychological state during competitions (Galli and Vealey, 2008). Coaches have specific tasks to facilitate the development and enhancement of athletes’ mental toughness. These tasks include creating a conducive environment, simulating competition-like conditions, elevating set goals, providing effective feedback, instilling confidence in athletes, and supporting their focus (Weinberg et al., 2016). Researchers have emphasized that understanding the concept of mental toughness could be beneficial for both coaches and athletes. Particularly, they have stated that with proper guidance from their coaches, athletes can enhance their performance by improving their motivation, focus, and ability to manage pressure when preparing for significant competitions (Middleton et al., 2004). From this perspective, mental toughness is not solely an internal psychological resource, but a socially embedded capacity shaped by communication patterns and feedback climates.

### 4.3 Factor 3: emotional regulation and sensitivity to evaluation

Participants in the third factor mentioned that they can remain calm and composed under pressure; however, they expressed concern about their coaches’ evaluations when they make mistakes. This mindset among the participants suggests that they are affected by negative evaluations when making mistakes and believe that this could have a detrimental effect on their performance. Reviewing the literature, there are various factors that can negatively impact athletes, such as intense training sessions, coach pressure, environmental conditions, and periods of intense training (Yılmaz, 2021). Coaches’ behaviors can influence athletes in various ways. Research indicates that coaches have a significant impact on athletes (Amorose and Anderson-Butcher, 2007; Van Exel and De Graaf, 2005). The communication style of the coach with the athlete can significantly affect the athlete’s psychological state and sporting performance (Cranmer and Myers, 2015). Athletes have stated that they are more motivated and perform better when they have positive communication with their coaches (West, 2016). Conversely, an aggressive communication style by the coach can lead to negative outcomes for athletes. Furthermore, it is noted that the coach’s negative communication style negatively affects the emotional state of athletes (Bekiari and Syrmpas, 2015).

### 4.4 Theoretical contribution and the role of Q methodology

A central contribution of the present study lies in its methodological approach. By employing Q methodology, the study reveals distinct subjective viewpoints that are often obscured in traditional scale-based research. Whereas variable centered approaches assume a shared latent structure of mental toughness, Q methodology captures how athletes organize and prioritize psychological attributes into coherent personal frameworks.

The research reveals varying views among athletes on significant issues such as their mental toughness skills and the impact of coach feedback on their performance. While the first group of athletes demonstrated a determined attitude towards overcoming challenges, the second group emphasized learning from mistakes and valuing their coaches’ feedback. On the other hand, the third group of athletes, while able to remain composed even under pressure, expressed concerns about the impact of their coaches’ evaluations on their performance. These findings underscore the importance of coaches effectively communicating with their athletes and providing support.

Importantly, these insights could not have been obtained through traditional scale-based approaches, which assume a shared latent structure of mental toughness. By contrast, Q methodology enabled the identification of distinct subjective viewpoints, capturing how athletes organize and prioritize psychological attributes in fundamentally different ways. This highlights the epistemological value of Q methodology in advancing theory development for complex and contested constructs such as mental toughness.

## 5 Practical suggestions and innovative approaches

Coaches should develop effective communication skills with athletes. In addition to providing positive feedback, they should empathize with athletes to understand and support their coping strategies for challenges.

For athletes aligned with Factor 1, applied interventions should emphasize structured goal setting, challenge-based training tasks, and problem-solving strategies that reinforce persistence under adversity. Each athlete has different needs. Coaches should work individually with athletes to support their strengths and identify areas for improvement.

For athletes aligned with Factor 2, coaching practices should prioritize timely, learning-oriented feedback following mistakes, as feedback is perceived as a central mechanism for restoring focus and performance. To support the process of learning from mistakes and development, coaches should provide constructive feedback focused on improving the athlete’s performance.

For athletes aligned with Factor 3, applied support should focus on managing evaluation-related stress through emotion regulation strategies and the development of psychologically safe coach athlete communication climates. To help athletes remain calm under pressure, coaches should teach stress management techniques and strengthen athletes’ coping skills. Coaches should help athletes learn and implement strategies to cope with performance anxiety. This can assist athletes in showcasing their best performance even in critical moments.

Implementing these recommendations can support athletes in both their personal development and achieving their best performance. However, it’s important to remember that each athlete-coach relationship is unique, and these recommendations should be applied considering individual needs and circumstances.

## 6 Strengths and weaknesses of the study

A limited number of studies examining the levels of mental toughness and cognitive skills of elite swimmers contributes to the strengths of our research. The use of Q methodology in this study provided an opportunity to deeply investigate the subjective experiences and opinions of elite swimmers. The applied method inherently aimed to elucidate specific and valuable insights. In addition to quantitative research methods, the focus was on individual differences in the concept of mental toughness. Designing the study with Q methodology is considered valuable due to its innovative approach in the field of sports psychology. The research provided an opportunity for athletes to express which factors affect their mental strength. These features of the study can be listed as its strengths.

The weakness of the study lies in the limited number of participants. Additionally, the lack of gender differentiation could be considered a weakness of the study.

## 7 Future research

The study focused on the factors influencing swimmers’ levels of mental toughness and classified individual opinions. In future research, cognitive processes associated with mental toughness should be investigated using the Q methodology, and practical solutions should be developed to improve cognitive processes in line with athletes’ opinions. Additionally, designing research with the Q method for different populations such as Olympic athletes and team sports players is believed to contribute to the sports psychology literature.

## Data Availability

The original contributions presented in the study are included in the article/supplementary material, further inquiries can be directed to the corresponding author.
